# Efficacy of a standardized cranberry supplement on overactive bladder symptoms and urobiome alterations in young women: a randomized, double-blind, placebo-controlled pilot trial

**DOI:** 10.3389/fcimb.2026.1952206

**Published:** 2026-10-07

**Authors:** Ayaka Ito, Shohei Akagawa, Yuko Akagawa, Shoji Tsuji, Shogo Kato, Sohsaku Yamanouchi, Kazunari Kaneko

**Affiliations:** 1 Department of Pediatrics, Kansai Medical University, Hirakata, Osaka, Japan; 2 Clinical Training Center, Kansai Medical University, Hirakata, Osaka, Japan

**Keywords:** cranberry, dysbiosis, *Lactobacillus*, overactive bladder, proanthocyanidins, randomized controlled trial, urobiome

## Abstract

**Introduction:**

Urobiome dysbiosis has been implicated in the pathogenesis of overactive bladder (OAB). In this study, the efficacy of a cranberry supplement for OAB symptoms was investigated, and concurrent urobiome alterations were evaluated in young women.

**Methods:**

This double-blind, randomized, placebo-controlled trial enrolled 26 women (median age, 23 years) with OAB (Overactive Bladder Symptom Score [OABSS] ≥ 3), who received either a cranberry supplement (36 mg proanthocyanidins per day) or placebo for 4 weeks. Clinical efficacy was assessed using the OABSS reduction rate, and urobiome composition was evaluated by 16S rRNA gene sequencing.

**Results:**

Twenty-five participants were included in the analysis. At 4 weeks, the OABSS reduction rate was greater in the cranberry group than in the placebo group (median, 66.7% vs. 40.0%; p = 0.039). At baseline, the urobiome was dominated by *Lactobacillus* (median relative abundance, 64.2%). After cranberry supplementation, the relative abundance of *Lactobacillus* decreased within the cranberry group (median change, −4.39%; p = 0.027; q = 0.096). No compositional changes were observed in the placebo group.

**Conclusions:**

Cranberry supplementation was associated with greater improvement in OAB symptoms than was observed with placebo in young women, while a decrease in *Lactobacillus* relative abundance was observed within the cranberry group. Given the small sample size of this exploratory pilot trial and the limited species-level resolution of 16S rRNA sequencing, these concurrent findings should be regarded as hypothesis-generating and warrant further investigation.

**Clinical trial registration:**

https://www.umin.ac.jp/ctr/, identifier UMIN000057544.

## 1 Introduction

Overactive bladder (OAB) affects 10–15% of adult women and can impair quality of life through urinary urgency, frequency, and nocturia (Irwin et al., 2006; Sadri et al., 2024). Behavioral therapy and pharmacotherapies (antimuscarinics or β3-adrenoceptor agonists) remain the standard treatment options (Cameron et al., 2024); however, the 12-month persistence rate for pharmacological treatment remains low at approximately 20% (Song et al., 2021). This poor adherence is largely attributable to adverse effects, including dry mouth and constipation, indicating an unmet need for safe, well-tolerated alternative therapies.

In this context, polyphenol-rich cranberries, particularly those containing proanthocyanidins (PACs), have attracted increasing interest. Although traditionally consumed for urinary tract infection prevention (Xia et al., 2021), recent evidence suggests that continuous cranberry intake may also alleviate OAB symptoms (Cho et al., 2021). However, evidence from high-quality randomized controlled trials (RCTs) remains limited, and the mechanisms underlying any therapeutic effect are unclear.

Recent evidence also indicates that urobiome composition differs between women with urinary tract conditions, including OAB, and healthy controls, suggesting that urobiome dysbiosis may be involved in OAB pathogenesis (Li et al., 2022; Palumbo et al., 2025).

Therefore, this double-blind, placebo-controlled RCT in young women aimed to evaluate the clinical efficacy of a standardized cranberry supplement for OAB symptoms. Additionally, intervention-associated urobiome alterations were evaluated to explore whether urobiome changes occurred in parallel with symptom improvement.

## 2 Materials and methods

### 2.1 Ethical considerations and trial registration

This study was conducted in accordance with the Declaration of Helsinki and was approved by the Ethics Review Committee of Kansai Medical University (approval number: 2024342). Written informed consent was obtained from all participants. The trial was registered with the UMIN Clinical Trials Registry (UMIN000057544).

### 2.2 Study design and participant recruitment

This was a single-center, double-blind, randomized, placebo-controlled, parallel-group pilot trial in young women with urinary urgency. To screen potential participants, an online survey (Google Forms) on OAB symptoms was administered to 724 female students enrolled in the Faculty of Medicine and the Faculty of Nursing at Kansai Medical University between November 27 and December 6, 2024. The survey collected data on age, height, weight, recent antibiotic use, and OAB symptoms based on the Overactive Bladder Symptom Score (OABSS) questionnaire (Homma et al., 2006). The OABSS is a validated symptom assessment tool comprising four questions on daytime frequency (score 0–2), nighttime frequency (score 0–3), urgency (score 0–5), and urgency incontinence (score 0–5), yielding a total score of 0 to 15, with higher scores indicating greater symptom severity. Responses were received from 375 students (response rate: 51.8%). Of these, 37 had an OABSS ≥ 3. Ten declined to participate, and one met an exclusion criterion because of pregnancy; the remaining 26 students provided written informed consent and were enrolled (**Figure 1**). A 4-week intervention was conducted between June and September 2025.

**Figure 1:**
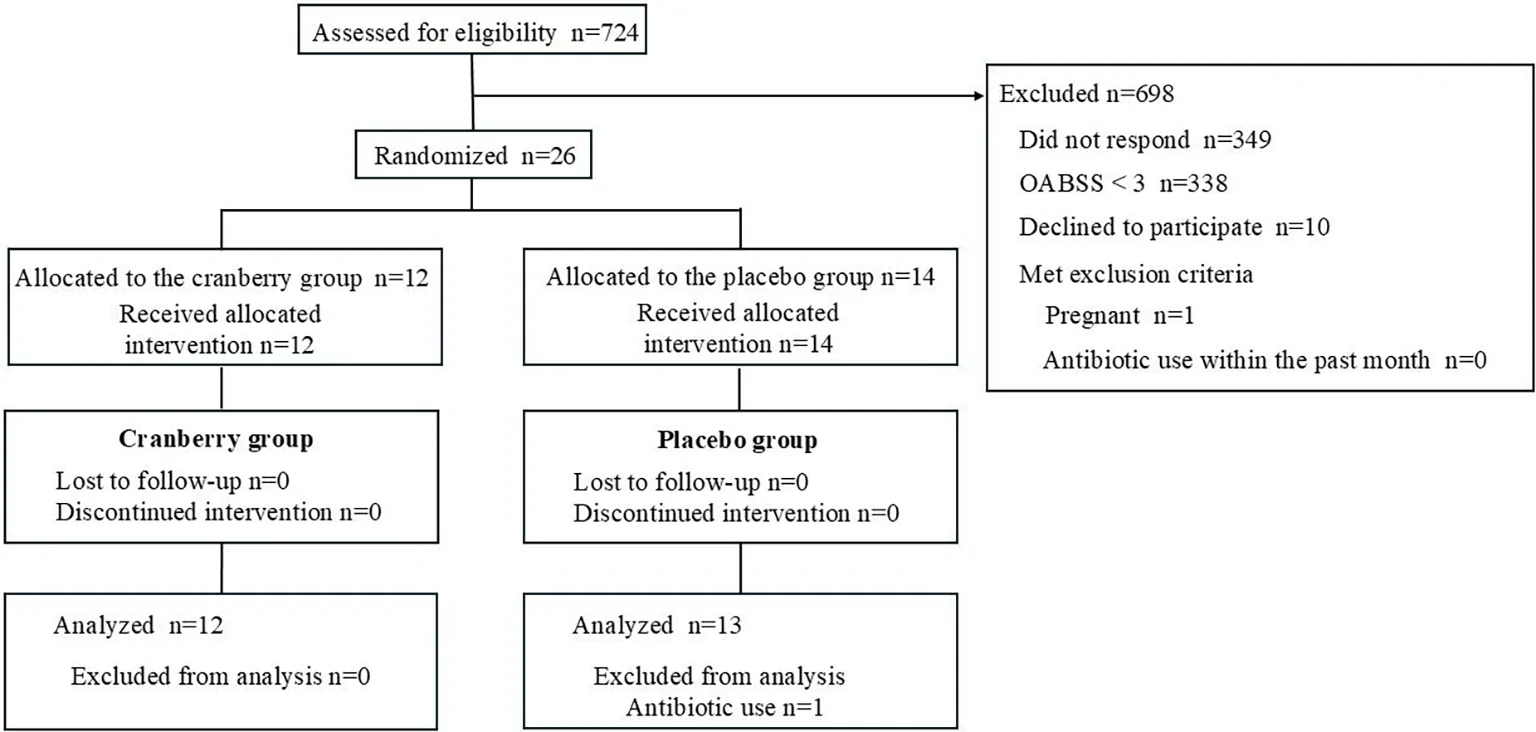
CONSORT flow diagram of the study participants. The flowchart shows the screening, enrollment, randomization, follow-up, and analysis of participants in this randomized, double-blind, placebo-controlled trial. OABSS, Overactive Bladder Symptom Score.

Female university students with an OABSS ≥ 3 were included in the study. The exclusion criteria were as follows: antibiotic use within the previous month, pregnancy, and a history of renal or urinary tract disease. Participants were withdrawn from the study if antibiotic use was reported during the study period, pregnancy was detected, they developed a renal or urinary tract disease, or the supplement adherence rate was <66%.

### 2.3 Randomization and blinding

The 26 enrolled participants were randomly assigned to the cranberry or placebo group. Allocation concealment was maintained using a centralized, web-based randomization system (UMIN INDICE cloud; UMIN Internet Data and Information system for Clinical and Epidemiological research). The investigators registered eligible participants in this system, which automatically generated treatment assignments, ensuring concealment of the allocation sequence until assignment. The placebo was designed to be indistinguishable from the cranberry supplement in appearance, taste, and odor. Both the investigators and participants were blinded to treatment allocation until database lock. Blinding adequacy was assessed at the end of the intervention by asking participants to guess whether they had received the cranberry supplement or placebo.

### 2.4 Intervention

The participants consumed five pellets (1 g) of either the cranberry supplement or a placebo once per day. Each 1 g cranberry dose was standardized to contain 36 mg of PACs, 33.1 mg of quinic acid, and 72.5 mg of polyphenols. Supplements were supplied at no cost by Marukai Corporation (Osaka, Japan) and were manufactured by Kobata Research Institute (Wakayama, Japan). The full list of inactive ingredients and the formulation process for both products is provided in the **Supplementary Material**.

### 2.5 Endpoints and follow-up

The primary endpoints of this study were the OABSS reduction rate (percentage decrease in score) from baseline to week 4 and changes in urobiome composition. Because low baseline scores were anticipated, the reduction rate was prespecified as the primary clinical endpoint to reduce floor effects and limit the influence of large absolute changes in a small number of participants. Urobiome changes were assessed using alpha diversity, beta diversity, and the relative abundances of bacterial genera accounting for ≥1% of the total composition. The secondary endpoint was the absolute change in OABSS. Adherence was monitored by collecting and counting unused supplement pellets returned at the end of the 4-week intervention.

### 2.6 Urine collection, DNA extraction, and 16S rRNA sequencing

At each assessment point, 10 mL of midstream urine was collected and frozen within 4 h of collection and stored at −80 °C until analysis. Genomic DNA was extracted from the urine pellet using the QIAamp DNA Micro Kit (QIAGEN, Hilden, Germany). The V3–V4 region of the 16S rRNA gene was amplified, and sequencing libraries were prepared and sequenced on the MiSeq i100 platform (Illumina, San Diego, CA). The detailed protocols for DNA extraction, PCR conditions, primer sequences, and library preparation are provided in the **Supplementary Material**.

### 2.7 Urobiome and statistical analyses

Alpha diversity (observed amplicon sequence variants [ASVs], Shannon, and Simpson indices) and beta diversity (Bray–Curtis dissimilarity, visualized by principal coordinate analysis [PCoA] and tested using permutational multivariate analysis of variance [PERMANOVA]) were assessed. Between-group comparisons of taxonomic composition were conducted at the genus level for taxa with ≥1% relative abundance. Statistical analyses were performed using JMP Student Edition 18 (SAS Institute Japan). Nonparametric data were analyzed using the Wilcoxon signed-rank test for paired groups and Mann–Whitney U test for independent groups; a two-*sided* p value < 0.05 was considered significant. Categorical variables were compared using Fisher’s exact test. Associations between the absolute change in OABSS and the change in *Lactobacillus* relative abundance were assessed using Spearman’s rank correlation coefficient. To account for multiple comparisons in the microbiome taxa analysis, the Benjamini-Hochberg false discovery rate (FDR) correction was applied. Given the exploratory nature of this pilot study, an adjusted p value (q value) < 0.1 was considered statistically significant. All statistical analyses were performed on a per-protocol basis and included the 25 participants who completed the trial according to the protocol. The appropriate sample size was estimated using G*Power 3.1.9.4. Based on a previous RCT demonstrating OAB symptom reduction (Cho et al., 2021), an effect size of 0.8, power of 0.8, and alpha error of 0.05 indicated a minimum of 21 participants per group. Because the enrolled sample size did not reach this required threshold, this study was considered an exploratory pilot trial.

## 3 Results

### 3.1 Participant characteristics, blinding, and safety evaluation

The 26 enrolled participants were randomly assigned to the cranberry (n=12) or placebo (n=14) group. Twenty-five participants were included in the per-protocol analysis; one participant in the placebo group was excluded because antibiotic use during the study met a predefined withdrawal criterion. The baseline characteristics of the participants are summarized in **Table 1**. The median age of the 25 participants included in the analysis was 23 (interquartile range [IQR]: 22–26) years, with no significant difference between the cranberry (23 [IQR: 23–25] years) and placebo (22 [IQR: 22–26] years) groups (p = 0.42). The median body mass index was also similar between the cranberry (20.8 [IQR: 19.9–22.5]) and placebo (20.5 [IQR: 20.2–21.4]) groups (p = 0.81). The median adherence rate to the study supplements was 88% [IQR: 82–93%] in the cranberry group and 93% [IQR: 82–97%] in the placebo group, with no significant difference between the groups (p = 0.41). No participant had an adherence rate below the withdrawal threshold of 66%. During the intervention period, no serious adverse events or dropouts due to supplement-related side effects were observed in either group. In the end-of-intervention blinding assessment, 44% of participants (11 out of 25) correctly identified their assigned product, which was consistent with maintenance of blinding.

**Table 1:** Baseline characteristics of the participants.

Characteristic	Total (n = 25)	Cranberry (n = 12)	Placebo (n = 13)	p value
Faculty (Medicine / Nursing)	15 / 10	8 / 4	7 / 6	0.69
Age (years)	23 (22–26)	23 (23–25)	22 (22–26)	0.42
BMI (kg/m²)	20.7 (19.9–21.9)	20.8 (19.9–22.5)	20.5 (20.2–21.4)	0.81
Total OABSS (0–15)	4.0 (3.0–5.0)	3.5 (3.0–5.3)	4.0 (3.0–5.0)	0.63

Data are presented as the median and interquartile range (IQR) or the number of participants. Between-group differences were assessed using the Mann–Whitney U test for continuous variables and Fisher's exact test for categorical variables. BMI, body mass index; OABSS, Overactive Bladder Symptom Score.

### 3.2 Baseline urobiome

16S rRNA gene sequencing of the urobiome yielded a median of 86,026 reads per sample (IQR: 76,643–93,481; minimum: 67,113). At baseline, urobiome composition did not differ significantly between groups (**Figure 2**). The cranberry and placebo groups did not differ in median alpha diversity, as measured by the number of ASVs (51.5 vs. 63.0), Shannon index (1.5 vs. 1.7), or Simpson index (0.48 vs. 0.54) (p = 0.48, 0.46, and 0.53, respectively). Beta diversity also did not differ significantly between groups (p = 0.59). Seven genera had an overall relative abundance of ≥1%: *Lactobacillus* (64.2%), *Bifidobacterium* (12.5%), *Prevotella* (6.0%), *Streptococcus* (5.1%), *Finegoldia* (1.4%), *Staphylococcus* (1.2%), and *Gemella* (1.2%); none differed significantly in relative abundance between groups. We also evaluated species-level composition within the predominant genus, *Lactobacillus*. Because of the limited taxonomic resolution of 16S rRNA sequencing, only 31% of *Lactobacillus* reads could be assigned to species. Among those assigned reads, *Lactobacillus iners* accounted for 99%.

**Figure 2:**
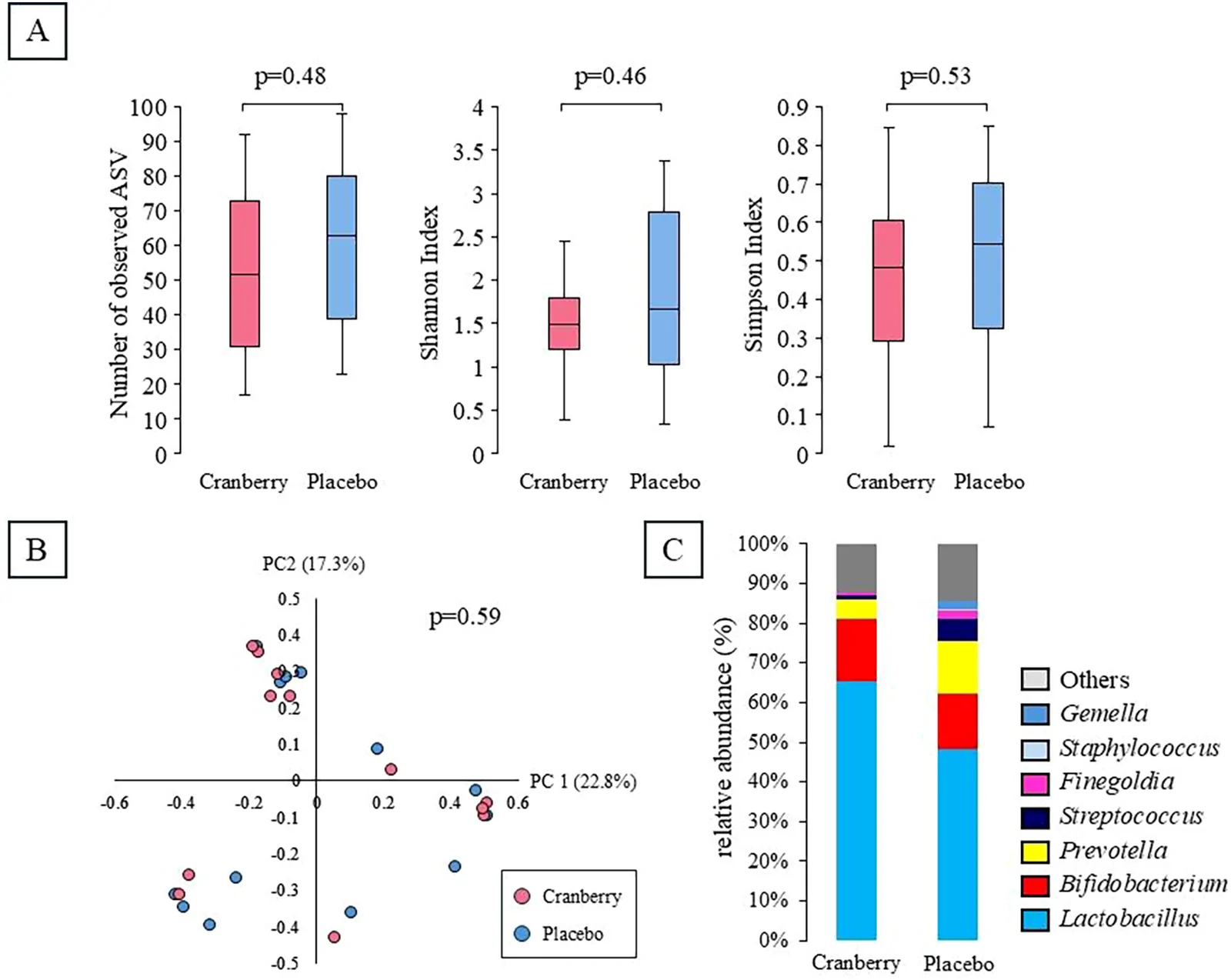
Baseline urobiome composition and diversity. **(A)** Alpha diversity indices (observed amplicon sequence variants [ASVs], Shannon index, and Simpson index) in the cranberry and placebo groups at baseline. **(B)** Relative abundance of bacterial genera accounting for ≥1% of the total urobiome. **(C)** Beta diversity visualized by principal coordinate analysis (PCoA) based on the Bray–Curtis dissimilarity index.

### 3.3 Changes in OABSS

The median baseline OABSS was 3.5 (IQR: 3.0–5.3) in the cranberry group and 4.0 (IQR: 3.0–5.0) in the placebo group, with no significant between-group difference (p = 0.63). After the 4-week intervention, OABSS significantly decreased from 3.5 to 1.5 (IQR: 1.0–3.0) in the cranberry group (p = 0.0001), and from 4.0 to 3.0 (IQR: 2.0–3.0) in the placebo group (p = 0.0026). Additionally, the median OABSS reduction rate (percentage decrease from baseline) was greater in the cranberry group than in the placebo group (66.7% [IQR: 41.7–70.0%] vs. 40.0% [IQR: 0–50.0%]; p = 0.039; confidence interval for difference in reduction rates: 1.3%−44.0%; effect size r = 0.35) (**Figure 3**). Furthermore, we also compared the absolute change in OABSS between the two groups. Although a trend toward a greater absolute reduction in the cranberry group was observed, this difference did not reach statistical significance (p = 0.14).

**Figure 3:**
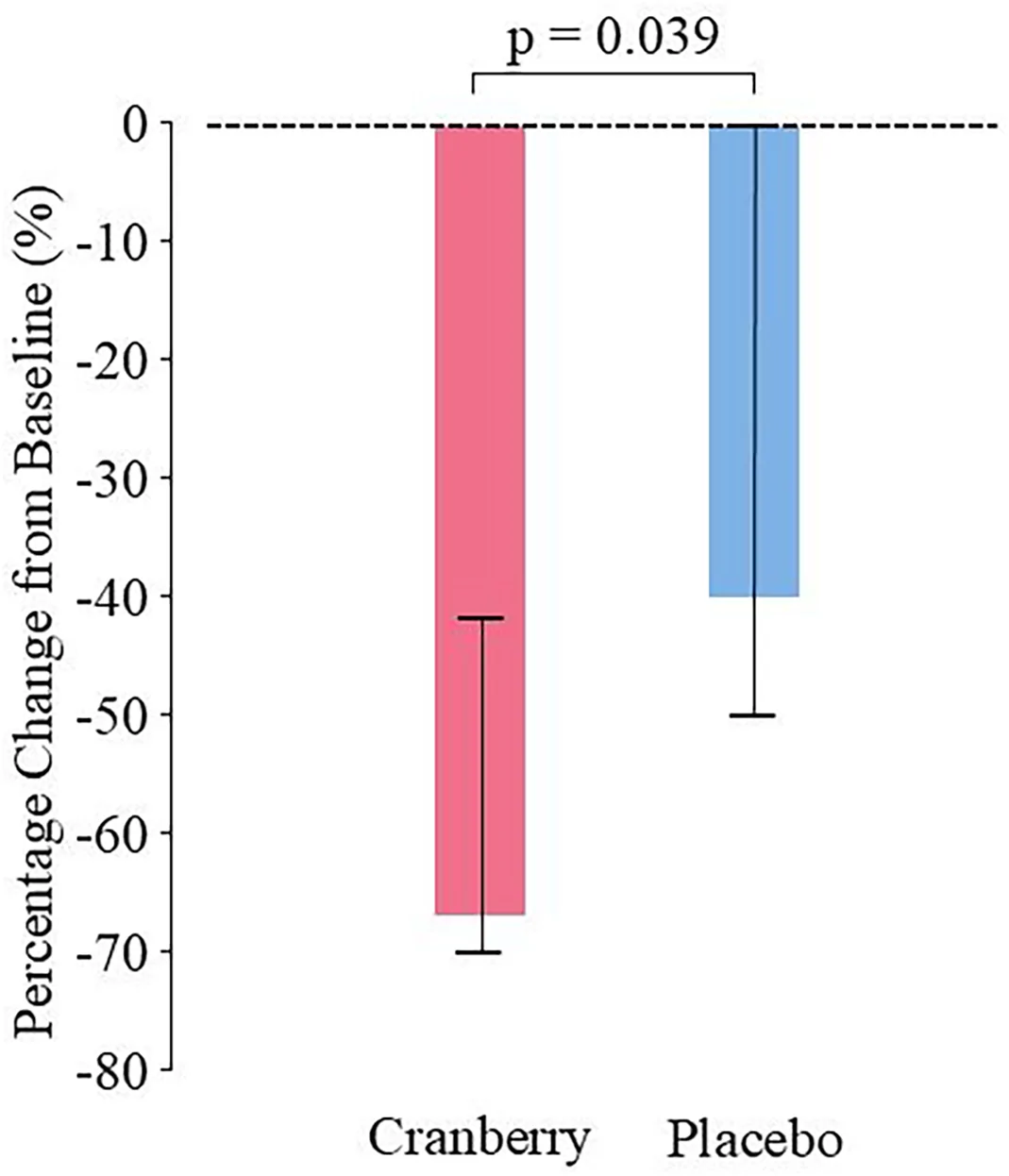
Changes in the overactive bladder symptom score (OABSS). Percentage reduction in OABSS from baseline after 4 weeks of intervention. The OABSS reduction rate was significantly greater in the cranberry group than in the placebo group (p = 0.039, Mann–Whitney U test).

### 3.4 Alterations in urobiome

Regarding alterations in the urobiome, no significant changes in alpha diversity were observed from baseline to week 4 in either the cranberry or placebo group (**Figure 4A**). In the cranberry group, the number of ASVs changed from 51.5 to 50.5 (p = 0.97), the Shannon index from 1.5 to 1.5 (p = 0.47), and the Simpson index from 0.48 to 0.43 (p = 0.34). In the placebo group, the number of ASVs changed from 63.0 to 61.0 (p = 0.54), the Shannon index from 1.7 to 2.0 (p = 0.38), and the Simpson index from 0.54 to 0.63 (p = 0.41). Similarly, no significant changes in beta diversity were observed after the intervention in either group (cranberry group: p = 0.99, placebo group: p = 0.99) (**Figure 4B**). After FDR correction of the seven genus-level comparisons, *Lactobacillus* decreased within the cranberry group (median change: −4.39% [IQR: −8.44 to 0.35]; p = 0.027; q = 0.096). *Bifidobacterium* showed a nominal increase before adjustment (median change: 0.05% [IQR: > −0.01 to 4.30]; p = 0.027), but this change was not statistically significant after FDR correction (q = 0.19) (**Table 2**). To assess the intervention effect directly, we compared the absolute change (Δ relative abundance) between the two groups. The between-group difference in the change in *Lactobacillus* was not statistically significant (p = 0.50). Spearman rank correlation analysis also showed no significant correlation between the absolute change in OABSS and the change in *Lactobacillus* relative abundance (Spearman’s *r_s_* = −0.029, p = 0.93).

**Figure 4:**
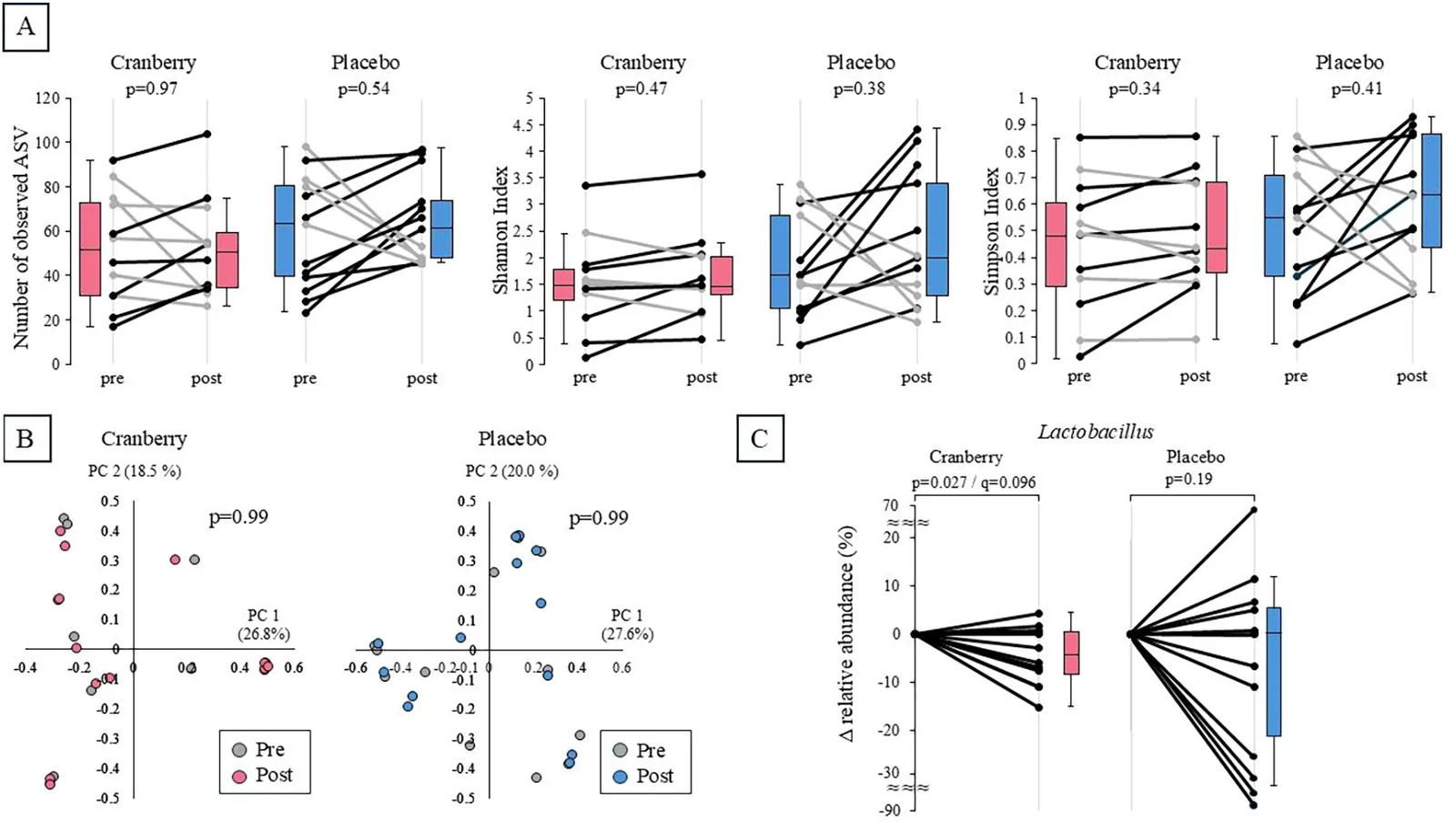
Alterations in the urobiome composition before and after the intervention. **(A)** Changes in alpha diversity indices (observed ASVs, Shannon index, and Simpson index) from baseline to week 4 in both groups. **(B)** PCoA plots showing beta diversity before and after the intervention. **(C)** Change in the relative abundance (Δ relative abundance) of *Lactobacillus* from baseline to week 4. The relative abundance of *Lactobacillus* significantly decreased in the cranberry group.

**Table 2:** Changes from baseline to week 4 in the relative abundance of major bacterial genera (≥1%).

Bacterial genus	Cranberry			Placebo		
	Δ relative abundance	p value	q value	Δ relative abundance	p value	q value
*Lactobacillus*	−4.39%	**0.027**	**0.096**	−0.03%	0.19	n/a
	(−8.44 to 0.35)			(−26.60 to 5.10)		
*Bifidobacterium*	0.05%	**0.027**	0.19	0.00%	0.50	n/a
	(> −0.01 to 4.30)			(−0.01 to 0.00)		
*Prevotella*	> −0.01%	0.43	n/a	0.02%	0.17	n/a
	(−0.02 to 0.01)			(−0.03 to 0.21)		
*Streptococcus*	> −0.01%	0.051	n/a	−0.01%	0.23	n/a
	(−0.01 to > −0.01)			(−0.14 to > −0.01)		
*Finegoldia*	> −0.01%	0.29	n/a	> −0.01%	0.45	n/a
	(> −0.01 to < 0.01)			(−0.01 to 0.01)		
*Staphylococcus*	< 0.01%	0.37	n/a	< 0.01%	0.26	n/a
	(> −0.01 to < 0.01)			(0.00 to < 0.01)		
*Gemella*	0.00%	0.25	n/a	0.00%	0.38	n/a
	(0.00 to 0.00)			(0.00 to 0.00)		

Data are expressed as the median and interquartile range (IQR). Statistical significance was assessed using the Wilcoxon signed-rank test. To represent non-zero, marginal fluctuations, positive values smaller than 0.01% are displayed as "< 0.01," whereas negative values with an absolute magnitude smaller than 0.01% are displayed as "> -0.01." n/a, not applicable. Values in bold indicate statistical significance (p < 0.05 or q < 0.1).

## 4 Discussion

This RCT evaluated the efficacy of a standardized cranberry supplement for alleviating OAB symptoms in 25 young women with an OABSS ≥ 3. Concurrent urobiome changes were also observed, with a decrease in the relative abundance of *Lactobacillus*.

To our knowledge, only one previous RCT by Cho et al. has investigated the efficacy of cranberry for OAB, reporting that daily intake of 500 mg of cranberry powder for 24 weeks significantly reduced micturition and urgency episodes (Cho et al., 2021). The significant OABSS improvement after cranberry supplementation observed in this study aligns with these earlier results.

This study differs from prior research in several key respects and provides additional insight. First, the cohort was limited to young women (median age, 23 years), whereas participants in the trial by Cho et al. had a mean age of approximately 48 years. This age-restricted cohort may have limited confounding factors more common in older adults, such as pelvic floor muscle weakening and lower female sex hormone levels. Second, the use of a standardized supplement containing 36 mg PACs, 33.1 mg quinic acid, and 72.5 mg polyphenols per daily dose may facilitate reproducibility of the intervention. Third, whereas the previous RCT evaluated a 24-week intervention, this study demonstrated significant OABSS reduction after only 4 weeks, suggesting relatively rapid symptom improvement. Finally, to our knowledge, this is the first RCT to report that symptom improvement with cranberry intervention occurs in parallel with urobiome compositional changes, extending beyond subjective clinical evaluation to explore potential underlying mechanisms.

Placebos can provide some symptom relief in OAB. A recent meta-analysis in Japanese patients with OAB reported that placebo administration significantly reduced urinary urgency and incontinence, with a pronounced effect emerging at 4 and 8 weeks (Hara, 2023). Such placebo responses are probably multifactorial. Keeping a regular voiding diary may encourage self-observation and act as behavioral therapy, whereas the expectation of receiving treatment can provide psychological reassurance (Mangera et al., 2011; Herschorn et al., 2017). In the present double-blind RCT, some improvement was observed in the placebo group (median reduction rate, 40.0%). Nonetheless, the cranberry group exhibited significantly greater improvement (median reduction rate, 66.7%), indicating a greater reduction in OABSS than that observed with placebo.

Regarding urobiome dysbiosis, previous reports in adult women with OAB have been inconsistent. Curtiss et al. and Wu et al. reported a significant decrease in the abundance of *Lactobacillus* in patients with OAB, whereas Javan et al. reported lower diversity and a higher relative abundance of *Lactobacillus*, particularly *L. iners*, compared with controls (Curtiss et al., 2017; Wu et al., 2017; Javan Balegh Marand et al., 2023). In this study, *Lactobacillus* also accounted for the highest compositional ratio in the baseline urobiome of young women with OAB (median 64.2%). Following cranberry intervention, this proportion significantly decreased, concurrently with OABSS improvement. Therefore, the dominance of specific *Lactobacillus* species may be associated with altered bladder homeostasis. Although *Lactobacillus* is generally considered protective in the urogenital tract, recent studies suggest that host immune responses to this genus vary by species. For example, Decout et al. reported that while *L. crispatus* interacts with DC-SIGN to induce immune tolerance, *L. iners* strongly activates Toll-like receptor 2 and may directly promote the production of inflammatory cytokines such as interleukin-8 from epithelial cells (Decout et al., 2024).

In our study, although species-level identification was limited to 31% of the total *Lactobacillus* reads, *L. iners* accounted for 99% of the reads assigned at the species level, indicating its dominance within the *Lactobacillus* community. We hypothesized that the *Lactobacillus* predominance observed in the bladder of young women with OAB may include species associated with micro-inflammation in the urothelium. The observed decrease in the abundance of *Lactobacillus* may be related to the anti-adhesion effects of cranberry polyphenols, which may physically inhibit bacterial colonization of the bladder mucosa. This cranberry-induced reduction aligns with our recent finding that continuous cranberry juice consumption decreases the relative abundance of *Lactobacillus* in the urobiome of healthy adult women (Akagawa et al., 2025). The consistency of these observations across cohorts is compatible with possible cranberry-associated urobiome modulation, although the underlying mechanism remains uncertain. Despite a decrease in *Lactobacillus* relative abundance within the cranberry group, the between-group difference in this change was not statistically significant (p = 0.50). The observed shift therefore cannot be attributed definitively to cranberry rather than placebo in this small cohort and should be interpreted as hypothesis-generating. Another potential mechanism is that bacterial metabolites may directly affect muscle tissue. Bacteria utilize quorum sensing (QS) to regulate gene expression by secreting autoinducers. A mechanistic study by De Spiegeleer et al. demonstrated that a QS molecule produced by a species of *Lactobacillus* directly induces the secretion of IL-6, an inflammatory cytokine, in myoblasts (De Spiegeleer et al., 2020). Such signaling could hypothetically contribute to bladder hypersensitivity or detrusor overactivity. However, because species-level resolution was limited to 31%, we cannot conclude that the overall decrease in *Lactobacillus* specifically reflects a reduction in *L. iners*. Together with the nonsignificant between-group comparison, this limitation leaves the proposed anti-adhesion and QS-related inflammatory pathways speculative. These mechanisms should therefore be viewed as a framework for hypothesis generation rather than as explanations of the present findings. Future adequately powered studies incorporating higher-resolution metagenomics, metabolomics, and concurrent profiling of urinary cytokines, chemokines, or other urothelial inflammation markers are needed to determine whether cranberry supplementation modifies the urobiome-inflammation-bladder symptom axis.

A slight nominal increase in the relative abundance of *Bifidobacterium* was observed after cranberry intake, but this finding did not remain statistically significant after correction for multiple comparisons. Although *Bifidobacterium* is known for its anti-inflammatory properties, any prebiotic effect of cranberry on this genus in the urobiome remains speculative. In the context of the potential local inflammatory signaling discussed above, this preliminary observation provides a rationale for future investigation rather than evidence of a biological effect. Future multiomics studies could examine whether such changes are associated with a shift toward a more anti-inflammatory microbial environment.

The major strength of this study is its randomized, double-blind, placebo-controlled design. However, several limitations must be acknowledged. First, the sample size (n=25) did not reach the target required for adequate statistical power for between-group comparisons. The study is therefore underpowered and should be interpreted as an exploratory pilot trial; both the clinical and urobiome findings are hypothesis-generating rather than confirmatory. The single-center setting and highly selected cohort also limit generalizability. Participants were exclusively young female university students (median age, 23 years) with relatively mild baseline OAB symptoms (median OABSS of 3.5 and 4.0), and therefore do not fully represent the broader, generally older population of patients with OAB, who may have more severe symptoms. Second, confounding factors, including the timing of the menstrual cycle and sexual intercourse, were not fully controlled, and 16S rRNA sequencing is limited in species-level identification (Price et al., 2020). Third, because this study involved otherwise healthy young women with OAB symptoms, midstream urine was collected for ethical reasons to avoid the invasiveness of catheterization. Combined with the absence of negative and blank controls for DNA extraction and PCR, use of midstream urine means that potential contamination from environmental, reagent, or other urogenital sources cannot be definitively excluded. Larger, multicenter studies incorporating higher-resolution shotgun metagenomic or metabolomic analyses are warranted to address these issues.

In conclusion, this pilot study found that daily intake of a standardized cranberry supplement was well tolerated and was associated with improvement in OAB symptoms in young adult women, with no serious adverse events observed during the 4-week intervention. Over the same period, a decrease in *Lactobacillus* relative abundance was observed within the cranberry group. These findings support further investigation of whether urobiome alterations are involved in OAB pathology in adequately powered confirmatory trials.

## Data Availability

The datasets presented in this study can be found in online repositories. The names of the repository/repositories and accession number(s) can be found below: https://www.ncbi.nlm.nih.gov/, PRJNA1499397.
