# Supplementary material for: Efficacy of a standardized cranberry supplement on overactive bladder symptoms and urobiome alterations in young women: a randomized, double-blind, placebo-controlled pilot trial
Source: Front Cell Infect Microbiol. 2026 Oct 7;16:1952206. doi: 10.3389/fcimb.2026.1952206 (PMC13683685; doi:10.3389/fcimb.2026.1952206)
Supplement: Supplementary file 1 [file SupplementaryFile1.docx]

***Supplementary Material***

**Supplementary Methods**

**Formulation of Supplements and Placebos**

The cranberry supplement comprised cranberry powder, concentrated cranberry juice, cranberry extract powder, glutinous rice, and cornstarch; these were mixed and granulated. The placebo consisted of high-quality rice flour, yam powder, shiratamako (refined rice flour), cornstarch, maltose, beet fiber, honey, and food coloring (Black A). The cranberry supplements and placebos were manufactured by Kobata Research Institute (Wakayama, Japan).

**Urine Collection and DNA Extraction**

At each assessment point, 10 mL of midstream urine was collected in a sterile container, and the samples were frozen within 4 h of collection and stored at –80°C until analysis. For DNA extraction, the samples were thawed, and a 2-mL urine aliquot was centrifuged at 14,500 rpm (20,000 × g) for 2 min to obtain a pellet. Genomic DNA was extracted from the pellet using the QIAamp DNA Micro Kit (QIAGEN, Hilden, Germany) following the manufacturer’s protocol. The extracted DNA was stored at –80°C until analysis.

**16S rRNA Library Preparation and Sequencing**

Sequencing libraries were prepared by amplifying the target region following the Illumina 16S Metagenomic Sequencing Library Preparation protocol. The first polymerase chain reaction (PCR) was performed using 5 or 10 ng of extracted genomic DNA as a template, 5× reaction buffer, 1 mM dNTP mix, 500 nM each of universal forward and reverse primers, and Herculase II Fusion DNA Polymerase (Agilent Technologies, Santa Clara, CA). The initial PCR conditions were as follows: initial heat activation at 95°C for 3 min, followed by 25 cycles at 95°C for 30 s, 55°C for 30 s, and 72°C for 30 s, with a final extension step at 72°C for 5 min. The universal primers with Illumina adapter overhang sequences used for the initial amplification, targeting the V3–V4 region of the 16S rRNA gene, were as follows:

forward primer: 5′-TCGTCGGCAGCGTCAGATGTGTATAAGAGACAGCCTACGGGNGGCWGCAG-3′; and reverse primer: 5′-GTCTCGTGGGCTCGGAGATGTGTATAAGAGACAGGACTACHVGGGTATCTAATCC-3′.

The first PCR products were purified using AMPure XP beads (Agencourt Bioscience, Beverly, MA, USA), and 10 μL of each purified products was subjected to a second PCR using Nextera XT Indexed Primers to construct the final index-containing libraries. The cycling conditions for the second PCR were identical to those of the first PCR, except that 10 cycles were performed. The second PCR products were purified with AMPure XP beads, quantified using the VICTOR Nivo™ system (PerkinElmer, Shelton, CT, USA) with PicoGreen reagents, and their quality was assessed using a TapeStation D1000 ScreenTape (Agilent Technologies). The libraries were quantified using quantitative PCR following the qPCR Quantification Protocol Guide (KAPA Library Quantification Kits for Illumina Sequencing Platforms), normalized, pooled, and sequenced using the MiSeq i100 platform (Illumina Inc., San Diego, CA).
